# Number and appraisal of daily hassles and life events in young adulthood: the association with physical activity and screen time: a longitudinal cohort study

**DOI:** 10.1186/1471-2458-14-1067

**Published:** 2014-10-13

**Authors:** Léonie Uijtdewilligen, Amika S Singh, Mai J Chinapaw, Lando LJ Koppes, Willem van Mechelen, Jos WR Twisk

**Affiliations:** Department of Public & Occupational Health and EMGO Institute for Health and Care Research, VU University Medical Center, Amsterdam, The Netherlands; Body@Work, Research Center Physical Activity, Work and Health, TNO-VU University Medical Center, Amsterdam, The Netherlands; Division Work and Employment, TNO, Hoofddorp, The Netherlands; Department of Health Sciences, Section Methodology and Applied Biostatistics, VU University Medical Center, Amsterdam, The Netherlands; Department of Epidemiology and Biostatistics, VU University Medical Center, Amsterdam, The Netherlands

**Keywords:** Adults, GEE, Prospective cohort, Sedentary behaviour

## Abstract

**Background:**

Young adults face radical life changes regarding residence, marriage, family and work that may negatively impact their health behaviours. Therefore, we investigated the associations of the number of daily hassles and life events and their subjective appraisal with physical activity and screen time in young adulthood.

**Methods:**

Data came from participants of the Amsterdam Growth and Health Longitudinal Study (AGAHLS). Self-reported physical activity (min/wk) was used from wave 6 (1991; mean age 27), wave 7 (1993; mean age 29), wave 8 (1996/1997; mean age 32) and 9 (2000; mean age 36). Self-reported screen time (h/wk) was assessed in waves 8 and 9. The number and the appraisal of daily hassles and major life events were assessed with the Everyday Problem Checklist and Life Events List, respectively (including five life event domains, i.e.: health, work, home/family, personal/social relations, and finances). The final sample included 474 participants for the physical activity analyses and 475 participants for the screen time analyses. To test the longitudinal associations of daily hassles and life events with physical activity and screen time, univariable and multivariable Generalised Estimating Equations were performed. Effect modification by gender was tested.

**Results:**

Physical activity levels were higher in those who had experienced more daily hassles. People who reported higher subjective appraisal in the work and finances life event domains also had higher levels of physical activity, although only the subjective appraisal in the finances domain remained significant in the multivariable model. No significant associations between number and subjective appraisal of daily hassles and life events and screen time were observed.

**Conclusions:**

The occurrence of *specific* life events may be more influential for people’s physical activity behaviour than their respective sum or emotional tone. Still, the assessment of daily hassles may be a relevant addition in this research field. Finally, we suggest that daily hassles and life events are less important for explaining screen time behaviour than for physical activity.

## Background

In modern societies, the median age for taking on the responsibilities of adulthood has shifted to the mid-twenties or early thirties [1]. Whereas previous generations had already reached stable life circumstances by the time they turned thirty, men and women around that age today still face radical life changes (or life events) regarding residence, marriage, family and work [1].

Results from a systematic literature review on the association between life events and change in leisure time physical activity, suggest that specifically in young adult women, leisure time physical activity decreases when they get a job or change work conditions, change from living alone to cohabiting, get married, or have a child [2]. Although results have been less conclusive for men, for both men and women it is assumed that experiencing multiple simultaneous events -which is not uncommon in young adulthood- has an adverse effect on physical activity participation [2]. Similar to physical *in*activity [3], sedentary behaviour (e.g., TV viewing, computer use, total screen time, motorised transport), is a health behaviour that is considered harmful for individual’s health [4]. Yet, life event-related research has not examined whether sedentary behaviour is associated with life events.

In contrast to the assumption that any life event no matter the emotional tone would negatively affect health outcomes [5], it is argued that the effects of life events vary depending on the meaning of the events to the individual [6]. In the Amsterdam Growth and Health Longitudinal Study (AGAHLS), young adults provided information on the number and the *subjective appraisal* of life events and reported their physical activity and screen time over several years [7]. Besides, life events that are usually considered as ‘minor’ (also referred to as daily hassles) like conflicts with colleagues, misbehaving children, being displeased about personal appearance, and being laughed at were assessed in the AGAHLS. Daily hassles have been examined seldom while they may have a greater influence on health and health-related behaviour than major life events [8] because they generate a constant source of stress.

The AGAHLS thus offers the opportunity to study the longitudinal associations between daily hassles, life events, physical activity and screen time in a young adult population, and as such address some of the gaps in life event-related research. The aims of the present study are therefore to examine the following:

1. What is the association between the number and the subjective appraisal of daily hassles and young adults’ physical activity behaviour and screen time?
2. What is the association between the number and the subjective appraisal of (different types of) life events and young adults’ physical activity behaviour and screen time?

## Methods

### Study design and participants

**Figure 1:**
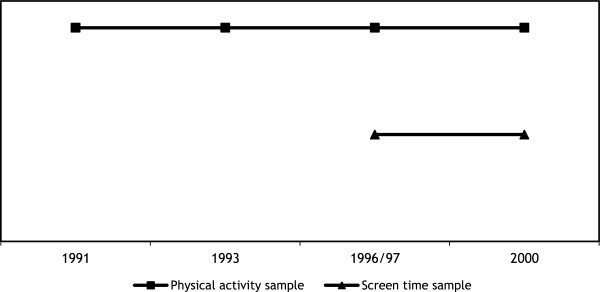
**Graphic presentation of the two samples that were used in this study.** Both dependent and independent factors were measured at the respective waves.

Data of this study come from the AGAHLS. This is an observational longitudinal study which started in 1976/1977 with monitoring growth, health, and lifestyle in approximately 600 13-year old healthy adolescents attending secondary school in the Netherlands. The study rationale, recruitment procedures and protocol have been reported in detail elsewhere [9, 10]. In the 30 years after the baseline measurement, nine measurement waves followed. The last (i.e., the 10th) measurement wave was conducted in 2006, at the age of 42 years. The AGAHLS was approved by the medical ethics committee of the VU University Medical Center, Amsterdam, the Netherlands. All subjects provided their written informed consent.In the AGAHLS, not all measures were consistently assessed at each wave. For the current study we therefore used two samples of subjects; one sample for physical activity and one for screen time (see Figure 1). The ‘physical activity sample’ included subjects who had provided data on life events, daily hassles and physical activity from wave 6 (1991) to wave 9 (2000) on at least one occasion. This sample comprised 474 subjects. Because screen time was only measured during wave 8 and 9, the ‘screen time sample’ included subjects who had provided data on life events, daily hassles and screen time from wave 8 (1996/1997) and wave 9 (2000) on at least one occasion. This sample comprised 475 subjects.

### Physical activity and screen time

Physical activity was measured by means of a semi-structured interview developed specifically for the AGAHLS [11]. Details on this measurement instrument have been reported before [12]. In brief, the interview covered the following activities over the past three months: organised sports, unorganised sports and other leisure-time activities, transportation to and from work, physical exertion at work, and stair walking. The total time spent on all these daily physical activities (min/wk) was calculated and used for the current analyses.

Screen time was assessed using the following questions:

1. When thinking about the previous three months, how many hours per week on average have you been watching TV during leisure: (i) during the week and; (ii) during the weekend?;
2. When thinking about the previous three months, how many hours per week on average have you spent behind the computer during leisure: (i) during the week and; (ii) during the weekend?

Total screen time (h/wk) was used for current analyses and was calculated by summing TV and computer time during week and weekend.

### Daily hassles and life events

Daily hassles were assessed using the Everyday Problem Checklist (EPC) [13], which covers 114 items referring to small, day-to-day irritations experienced during the past two months. Participants rated each item on a 4-point scale ranging from 0 (‘I do not mind at all’) to 3 (‘I do mind a lot’). From the EPC, two scores were derived i.e., (1) the total number of daily hassles experienced and (2) a summation of the individual item scores, reflecting the total subjective appraisal of daily hassles.

The EPC was shown to have a good test-retest reliability for number (r = .87), intensity (r = .85) and total score (r = .85), and was found to be moderately positive associated with distress in a general population [14]. Life events were assessed using the Life Events List (LEL) - a translated (into Dutch) version of the Life Events Survey of Sarason, Johnson and Siegel [15]. Test-retest reliability of the original LEL was evaluated, and the LEL was found to be a moderately reliable instrument for assessing life events [15]. The LEL covers 90 possible life event items in five domains i.e., health (8 items), work (15 items), home/family (38 items), personal/social relations (23 items), and finances (6 items). Participants rated each item on a 7-point scale ranging from 1 (very positive) to 7 (very negative), and were asked to indicate life events experienced during the past year. From the LEL, two main scores were derived i.e., (1) a score based on the total number of life events experienced, and (2) a score which expressed the total subjective appraisal of experienced life events. Not applicable life events and events that were experienced as ‘having no influence’ were scored 4. For each of the 5 domains, we calculated the total number of experienced life events, and participants’ subjective appraisal. As the created EPC and LEL scores had either small or wide response ranges, for each variable one step on the scale was defined as 10% of the total scale range of that respective variable.

### Statistical analysis

To study longitudinal associations of daily hassles and life events with physical activity and screen time, we first conducted linear Generalised Estimating Equations (GEE) analyses with daily hassle and life event parameters as independent and physical activity and screen time as dependent variables. Subsequently, we performed multivariable GEE analysis including all independent variables, excluding the total number and the subjective appraisal of experienced life events. Independent variables with the highest *p*-value were removed from the model stepwise until only variables with *p* < .05 remained. All analyses were adjusted for age and gender. In addition, we evaluated possible effect modification by gender (*p* < .05). As physical activity levels were not normally distributed, a successful log transformation was performed. Screen time was normally distributed, and so the analyses were performed without log transformation of screen time. All statistical analyses were conducted with the Statistical Package of Social Sciences, 18.0 for Windows (SPSS, inc., Chicago, Illinois, USA). Results were considered significant if *p* < .05.

## Results

Table 1 shows descriptive information for AGAHLS participants regarding the number and appraisal of experienced daily hassles and life events. The number of daily hassles peaked at the age of 36, while the number of life events was highest at age 27. The negative appraisal of daily hassles was considerably higher at age 36 than before, while for life events the subjective appraisal remained relatively stable over time.

**Table 1:** **Descriptives of gender, life events, daily hassles, physical activity and screen time in AGAHLS participants**

Calendar age in years (year of measurement)	27 (1991)	29 (1993)	32 (1996/1997)	36 (2000)
	n = 180	n = 164	n = 431	n = 400
Gender (% men)	46	47	47	47
**Daily hassles**				
Total number of daily hassles during the past two months				
Median	17	15	16	26
Overall range	0-114	2-71	1-114	0-92
Total appraisal of daily hassles during the past two months				
Median	21	18	19	27
Overall range	0-112	1-158	0-112	0-142
**Life events**				
Total number of life events during the past year				
Median	16	12	12	11
Overall range	0-86	0-33	0-89	0-41
Total appraisal of life events during the past year				
Median	349	355	356	356
Overall range	315-419	287-382	310-443	314-409
**Physical activity (min/wk)**				
Median	399	376	427	657
Interquartile range	417	327	382	620
**Screen time (h/wk)**				
Mean(sd)			16(9)	17(9)

The most frequently reported life events in the health domain across all ages included; change in usual type and/or amount of leisure time recreation, illness, and change in sleeping habit (much more or much less). In the work domain, more responsibility at work, change in working hours or circumstances and difficulties at work/reorganisation were most frequently reported from age 27 to age 36 years. In the home/family domain, change in living circumstances, an increase or decrease in the number of disagreements with partner, and change in the occurrence of family gatherings were experienced most over the nine year follow-up. At age 32 and 36 a ‘change in health of parent’ was also frequently reported. The most frequently reported life events in the personal/social domain included; holiday’s, change in social activities, realisation of an important personal goal, and making an important decision concerning the near future. Finally, in the financial domain, improvement in one’s financial situation, realising a long-saved purchase, and lending a large amount of money were most frequently reported across all ages.

### Daily hassles, life events and physical activity

Results from the univariable models showed that participants who had experienced more daily hassles had higher levels of physical activity (Table 2). Also, those with higher appraisal in the work and finances life event domains had higher levels of physical activity (Table 2).

**Table 2:** **Univariable associations** ^**a**^ **of daily hassles and life events with physical activity and screen time**

	Physical activity		Screen time	
	Effect ^b^	(95% CI)	B	(95% CI)
**Daily hassles (score on EPC)**				
Total number	1.02***	**(1.01;** 1.03)	-.03	(−.42; .36)
Total appraisal	1.00	(.99; 1.02)	-.16	(−.51; .17)
**Life events (score on LEL)**				
Total number^c^	1.00	(.99; 1.01)	.22	(−.13; .57)
Number health domain	.99	(.98; 1.00)	.20	(−.02; .42)
Number work domain	.99	(.98; 1.00)	-.02	(−.36; .32)
Number home/family domain	1.00	(.99; 1.01)	.11	(−.24; .47)
Number personal/social relations domain	1.00	(.99; 1.01)	.21	(−.14; .56)
Number finances domain	1.00	(.99; 1.01)	.09	(−.17; .35)
Total appraisal^c^	1.00	(.99; 1.01)	.33	(−.19; .87)
Appraisal health domain	1.00	(.89; 1.01)	.27	(−.15; .70)
Appraisal work domain	1.01**	**(1.00;** **1.02)**	.15	(−.22; .53)
Appraisal home/family domain	1.00	(.99; 1.01)	.32	(−.18; .83)
Appraisal personal/social relations domain	.99	(.98; 1.01)	.03	(−.36; .43)
Appraisal finances domain	1.01**	**(1.00;** **1.03)**	.17	(−.28; .62)

EPC = Everyday Problem Checklist; LEL = Life Events List.

^a^Univeriable analyses were corrected for age and gender.

^b^Values higher than 1 represent a higher (%) of physical activity level for each additional step (10%) on the determinant scale.

^c^Excluded from the multivariable model

Bold values represent statistically significant results; **p < .01; ***p < .001.

After running the multivariable analyses, having experienced more daily hassles (effect = 1.02, 95% CI 1.01-1.04, *p* < .001) and a higher appraisal in the finances domain (effect = 1.01, 95% CI 1.00-1.02, *p* = .04) remained significantly associated with higher levels of physical activity (data not shown). This means that with every 10% increase on the number of hassles scale and the financial appraisal scale, an individual’s time spend in physical activity (min/wk) increases with 2% and 1% respectively.

### Daily hassles, life events and screen time

Results from the univariable models showed no significant associations between number and subjective appraisal of daily hassles and life events and screen time (Table 2). Also, in the multivariable model none of the independent variables were significantly associated with screen time.

No significant effect modifications by gender were found, meaning that the association between daily hassles, life events and physical activity and screen time, respectively, were not significantly different for men and women.

## Discussion

The current study examined the longitudinal associations between the number and the subjective appraisal of daily hassles and life events, and young adults’ physical activity levels and screen time.

Young adults who had experienced more day-to-day irritations over the past months, were also more physically active during that period. Those who had higher subjective appraisal concerning work and finances-related life events over the past year were more physically active, however, when combined in one model, only the finances-related life events remained significant.

### Physical activity

A systematic literature review examining the associations between life events and physical activity, concluded that the occurrence of specific major life events (e.g., getting married, changing work conditions, interpersonal loss, retirement) either decreased or increased adults’ participation in leisure time physical activity [2]. Yet, only three studies that were included in this review [16–18] assessed the association between the *number* of life events and physical activity. One of these studies also examined AGAHLS data [16], taking into account the 1996/1997 measurement only. Notably, unlike the current study, two of the three aforementioned studies included adults with a mean age over 50 years [17, 18]. Based on these studies it was concluded that experiencing multiple simultaneous life events decreased physical activity in both men and women [2]. Our findings do not support this conclusion. This may be due to the differences in age between our population and the populations previously examined, but there is obviously a lack of studies that investigated the association between the number of life events and physical activity levels in general.

With regard to the potential influence of the *appraisal* of life events on physical activity, the review of Engberg and colleagues [2] provided no information, and neither did another systematic literature review on the association between life-change events (i.e., change in: employment status, residence, physical status, relationships and family structure) and physical activity participation by Allender et al. [19]. Our study showed that those who reported higher negative appraisal in the work and finances life event domains had higher levels of physical activity, but due to the lack of comparable studies and the operationalization of the life events measures we used (see also Strengths and limitations), we cannot suggest an explanation for these findings.

The difference between life events and daily hassles is that the latter are inconvenient in nature. All items of the EPC were formulated such that no person could possibly assess them in a positive way (e.g., ‘you had to make a difficult decision’ , ‘your children misbehaved’ , ‘people you thought you could count on disappointed you’). Considering that a) daily hassles may cause a certain level of distress [14], and b) previous research has indicated that physical activity can influence the perceived stress level and thus can be beneficial for psychological wellbeing [20], young adults from our sample might have used physical activity in order to reduce strain from day-to-day irritations. Already in the 1980s it was thought that chronic strains of everyday life were more strongly associated with health outcomes than major life events [8, 21]. This was also the case in our study considering the respective ‘effects’ and *p*-values. Daily hassles may thus be more proximal to individual health and health-related behaviour and could therefore be an important addition to life event research in the field of physical activity.

### Screen time

With regard to screen time, we found no significant associations with daily hassles and life events (both number and appraisal). One may put forward that this could be due to the cumulation of TV viewing and computer use as both screen-based behaviours may have different purposes; the first for relaxation or distraction, the second for work or maintaining social contacts. Yet, when assessing associations between daily hassles, life events and screen time for TV viewing and computer use separately, no significant findings were observed either. Based on our findings, we think that daily hassles and life events are less important for explaining screen time behaviour than for physical activity. Since, to our knowledge, this is the first study that examined these associations more research is needed to confirm our findings.

### Strengths and limitations

In our study, positive and negative life events were operationalized into one sum score (number), as was the positive and negative appraisal. We hypothesized that the results of positive life events on physical activity or screen time would be ruled out by negative life events. For smoking, such adverse effects of negative life events are well established [22, 23]. Yet, as physical activity and screen-based behaviours are in essence very different from smoking, one could argue that both negative and positive appraised life events are differently associated with either behaviour. Splitting up the number and appraisal of negative and positive life events did not result in different findings in this particular Dutch young adult population. In our opinion, examining the number and appraisal of multiple life events is complex, as negative life events may increase physical activity behaviour in some people, but decrease physical activity in others. And this hypothesised mechanism holds for positive life events as well. Besides, each domain we have investigated consisted of grouped life events of which some were likely to be positively appraised (e.g., holiday in the personal/social domain), and some were likely to be negatively appraised (e.g., conflict with the law that led to imprisoning in the personal/social domain). From a health perspective, it may be more interesting to know how the number and appraisal of *specific* life events are associated with physical activity or screen time than to assess the overall association with negative versus positive life events or their clustering in diverse domains.

Our study extends earlier findings by its longitudinal nature and the assessment of associations of life events and daily hassles with screen time. Still, this study included a three month self-reported recall of physical activity and screen time which may suffer from recall bias and self-report errors [24, 25]. Generalisation of the results to the general Dutch or to other populations may be difficult, since AGAHLS participants were relatively healthy and were of relatively high socio-economic status.

## Conclusion

Based on our findings and considering the consistent evidence from life event-related research, the occurrence of *specific* life events may be more influential for people’s physical activity behaviour than their respective sum or emotional tone. Still, the assessment of daily hassles may be a relevant addition in this research field. Finally, we think that daily hassles and life events are less important for explaining screen time behaviour than for physical activity.

## Abbreviations

AGAHLS: Amsterdam growth and health longitudinal study
EPC: Everyday problem checklist
LEL: Life events list
GEE: Generalised estimating equations.
